# Proteomic research and diagnosis in bladder cancer: state of the art review

**DOI:** 10.1590/S1677-5538.IBJU.2021.99.02

**Published:** 2020-05-10

**Authors:** Jorge Luis Wilson, Mariana Pereira Antoniassi, Paula Intasqui Lopes, Hatylas Azevedo

**Affiliations:** 1 Universidade Federal de São Paulo Divisão de Urologia Departamento de Cirurgia São PauloSP Brasil Departamento de Cirurgia, Divisão de Urologia, Universidade Federal de São Paulo - UNIFESP, São Paulo, SP, Brasil.

**Keywords:** Urinary Bladder Neoplasm, Proteomics, Biomarkers

## Abstract

**Purpose:**

Proteomic biomarkers have been emerging as alternative methods to the gold standard procedures of cystoscopy and urine cytology in the diagnosis and surveillance of bladder cancer (BC). This review aims to update the state of the art of proteomics research and diagnosis in BC.

**Materials and Methods:**

We reviewed the current literature related to BC research on urinary, tissue, blood and cell line proteomics, using the Pubmed database.

**Findings:**

Two urinary protein biomarkers are FDA-approved (NMP22® and BTA® tests), only if performed along with cystoscopy for surveillance after initial diagnosis, but not in the primary diagnostic setting due to high false-positive rates in case of infections, stones and hematuria. There are a great number of non-FDA approved proteins being studied, with good preliminary results; panels of proteins seem valuable tools to be refined in ongoing trials. Blood proteins are a bigger challenge, because of the complexity of the serum protein profile and the scarcity of blood proteomic studies in BC. Previous studies with the BC tissue proteome do not correlate well with the urinary proteome, likely due to the tumor heterogeneity. Cell line proteomic research helps in the understanding of basic mechanisms that drive BC development and progression; the main difficulty is culturing low-grade tumors in vitro, which represents the majority of BC tumors in clinical practice.

**Conclusion:**

Protein biomarkers have promising value in the diagnosis, surveillance and prognostic of BC. Urine is the most appropriate body fluid for biomarker research in BC due to its easiness of sampling, stability and enrichment of shed and secreted tumor-specific proteins. Panels of biomarkers may exhibit higher sensitivity than single proteins in the diagnosis of BC at larger populations due to clinical and tumor heterogeneity. Prospective clinical trials are warranted to validate the relevance of proteomic data in the clinical management of BC.

## INTRODUCTION

Bladder cancer (BC) represents the 9th most common cancer in the World. In Brazil, 9.480 new cases were estimated in 2018 (1, 2). The most common histology is the transitional cell carcinoma, accounting for about 95% of cases. The majority of the patients are diagnosed at an early stage and with localized disease, with approximately 70-80% having non-invasive disease (Ta, T1, Tis), and the remaining 20-30% presenting the muscle invasive disease subtype (3). Non-invasive tumors have a relapse risk of 31% to 78% within 5 years of diagnosis in the low-risk and high-risk subgroups, respectively, whereas progression risk ranges from 0.8% to 47%, respectively (4).

Despite the advances in new biomarkers, the gold standard for BC diagnosis and follow-up is still based on cystoscopy and urinary cytology (5). Cytology has high specificity (80-90%) but low sensitivity (25-48%), especially for low-grade tumors (6). Consequently, the definitive diagnosis depends on the identification of the bladder lesion after cystoscopy and biopsy, an invasive and expensive method that usually requires hospitalization (7).

Novel molecular biomarkers have been explored for the noninvasive surveillance of non-muscle invasive bladder cancer (NMIBC), with the potential to minimize the number of control cystoscopies, and to offer a lower-cost approach for cancer monitoring. In the high-risk patient subgroup, the differentiation between NMIBC and muscle-invasive bladder cancer (MIBC) is also of great interest, because it can modify or intensify the surveillance protocol (8). Besides that, new molecular biomarkers may also play an important role as predictors of treatment response (9) with potential better accuracy than clinical or cellular parameters described so far, such as the prognostic value of skeletal muscle index after radical cystectomy (10), or the neutrophil-to-lymphocyte ratio for assessing the response to intravesical treatment with BCG (11). Therefore, this review aimed to update the state of the art of proteomics research in BC, focusing on urinary, cancer tissue, blood and cell lines proteomics studies.

## MATERIALS AND METHODS

We reviewed the current literature that investigated the BC proteome in urine, tissue, blood and cell lines. Relevant articles were searched in the Pubmed database (http://www.pubmed.gov), using the key words “bladder cancer”, “urotelial carcinoma”, “urinary proteomics”, “tissue proteomics”, “blood proteomics” and “cell lines proteomics”, in several combinations. Only articles written in English were included.

### Urinary proteomics

Urinary proteins are composed of plasma proteins after they pass through the glomeruli, as well as of those secreted by the urothelium itself. Urinary proteomic studies have identified candidate biomarkers for urogenital diseases, such as acute kidney injury and bladder cancer (12-14). The direct contact with the bladder tumor makes the urinary proteome a prominent clinical source for the search of biomarkers, since it can directly reflect the BC biology due to the presence of specific proteins that could represent the tumor molecular phenotype at a certain moment (15, 16). Moreover, the analysis of urine samples is a non-invasive method that is easier than blood to sample; these atributes have established urine as a good source of urinary biomarkers for BC diagnosis and surveillance. We list below a brief review of the main urine protein biomarkers.

### FDA-approved urine protein biomarkers

The Food and Drug Administration (FDA) has approved the use of five urine-based tests commercially available. Three of them detect DNA, RNA or protein changes in urinary cells (UroVysion® Fluorescent In Situ Hybridization, Cxbladder™ and ImmunoCyt™), and the other two quantify proteins released into the urine (NMP22® and BTA® tests) (9, 17). The UroVysion® FISH test detects the aneuploidy of chromosomes 3, 7 and 17, and loss of both 9p21 loci in malignant urothelial cells, showing a diagnostic sensitivity of 87.3% and specificity of 71.4% (18). Cxbladder™ measures the urine mRNA levels of five cancer biomarkers, and combines this information with clinical variables to generate a final binary classification with a sensitivity of 85% and specificity of 82% (19). ImmunoCyt™ is an immunocytochemical test that uses fluorescent-labeled antibodies to detect three proteins commonly found in malignant urothelial cells: glycosylated carcinoembryonic antigen and two less glycosylated mucins. The sensitivity of ImmunoCyt™ ranges from 38.5% to 92.1% across all tumor grades and is generally more sensitive than standard voided cytology. However, the specificity (62% to 84.2%) of ImmunoCyt™ is inferior to cytology (20).

The NMP22® test is available with the comercial name of NMP22 Bladder Chek®, (Alere, Waltham, Mass., USA). It detects the nuclear mitotic apparatus protein 1 (NUMA1), also called Nuclear matrix protein-22 (NMP22). This protein is released by apoptotic cells into the urine, and is 25-fold overexpressed in BC cells compared to benign urothelium cells (21). The test is approved for surveillance after initial treatment and for early diagnosis in high-risk cases. Its sensitivity and specificity vary depending on the number of lesions and the tumor grade. Besides the volume of tumor tissue, tumor grade is an additional variable that influences NMP22 levels, with better sensitivity seen for high-grade tumors (22). For the quantitative NMP22 test, sensitivity is around 69% and specificity around 77%, while for the qualitative NMP22 test sensitivity is around 58% and specificity 88% (23, 24). NMP22 has a high rate of false-positive in cases of infections, stones, hematuria or previous instrumentation, with lower specificity compared to urine cytology (25). Although it has a high sensitivity, the high rate of false positive tests represents the most important limitation for its implementation in routine NMIBC management.

The bladder tumor antigen test (BTA® test) identifies a basement membrane antigen related to human complement factor H that is increased in the urine of BC patients. It is also available in both qualitative (BTA stat®) and quantitative (BTA TRAK®) formats. The BTA stat® test is a single step, qualitative immunochromatographic assay for the detection of bladder tumor associated antigen. This protein protects BC cells from complement-mediated lysis (26). The BTA TRAK® is a quantitative test that is more sensitive than the BTA stat® (27). Both tend to show false positives in the presence of inflammation, hematuria and other genitourinary malignancies, resulting in lower specificity than urine cytology. Their sensitivity and specificity are around 65% and 75%, respectively (23, 28). The FDA has approved both tests, but only as a companion diagnostic tool with cystoscopy (29).

Despite the approval of these tests by the FDA, they have not yet been incorporated into the American Urological Association or in the European Association of Urology clinical guidelines for BC diagnosis and surveillance (30).

### NON-FDA Approved urine protein biomarkers

Novel urinary protein biomarkers have been studied to complement the clinical information provided by the above-mentioned FDA-approved tests. Table-1 displays a summary from the literature with the most promising novel urinary protein biomarkers. Relative differences in expression of several proteins such as keratins have been observed when comparing normal urothelium to BC. Urothelial cytokeratins are predictors of cell death that can be released into the urine in the presence of cancer. Cytokeratin 8 (KRT8) and 18 (KRT18) are the most studied ones, detected by immunological assays (ELISA), with sensitivity ranging from 50 to 61% and specificity ranging from 63 to 97%, but with high false-positive rates, being positive in many epithelial cancers, and limited sensibility to low-grade tumors (31-33). The Urinary Bladder Cancer assay (UBC-enzyme-linked immunosorbent assay, IDL Biotech, Borlange, Sweden) is a test that evaluates cytokeratins 8 and 18 in the urine from urothelial tumors, and some studies suggested it performed better than BTA stat test and NMP22 (34). Cytokeratin 20 (KRT20) urinary expression, evaluated by reverse transcription (RT)-polimerase chain reaction (PCR) assay, shows 78 to 87% sensitivity and 56 to 80% specificity, but poor sensitivity for low-grade tumors (35).

**Table 1:** Non-FDA Approved potencial urinary protein biomarkers for bladder cancer detection.

Proteins	Reference	Subjects	Sensivity	Specificity
Cytokeratin 8 and 18 (KRT8 and KRT18)	Mian et al. 2000 (34)	54 histologically UCC, 186 controls	65%	92%
Cytokeratin 20 (KRT20)	Mi et al. 2018 (35)	2291 BC cases, 1182 controls	79%	90%
Bladder cancer markers 1 (BLCA-1)	Myers-Irvin et al. 2005 (39)	25 BC patients, 46 controls	80%	87%
Bladder cancer markers 4 (BLCA-4)	Konety et al. 2000 (37)	54 BC patients, 51 controls	89-96%	90-100%
Aurora A kinase (AURKA)	de Martino et al. 2015 (40)	122 BC patients, 66 controls with hematuria	83.6%	65.2%
Panel of 22 urinary proteins	Theodorescu et al. 2006 (43)	31 BC patients, 11 healthy individuals, and 138 patients with non-malignant genitourinary disease	100%	73%
4 urinary biomarkers (PGRMC1, COL1A1, UMOD and COL3A1)	Schiffer et al., 2009 (44)	71 NMIBC and 56 MIBC patients, 297 controls	92%	68%
116 urinary proteins	Frantzi et al, 2016 (45)	341 BC patients and 110 controls	91%	68%

BLCA-1 and BLCA-4 (bladder cancer markers 1 and 4) are two transcription factors that are found in early stages of BC tissues, even before the appearance of a visible tumor (28), and have no interference with infection, smoking, catheterization or cystitis (36). BLCA-4 is expressed in the normal urothelium of a bladder containing tumor, but not in bladder tissue unaffected by cancer. This was demonstrated in a study where 51 normal tested individuals were negative for BLCA-4 expression, whereas 53 out of 55 samples from patients with BC were positive. Its sensitivity ranges from 89 to 96% and specificity from 90 to 100% (37, 38). BLCA-1 is detectable in urine from patients with BC by immunoassays. It is also detectable in tumor tissue, but not in normal adjacent areas or in bladder tissue from healthy donors. It was detected in 20 of 25 urine samples from BC patients, but only in 6 of 46 normal, high risk, prostate or renal cancer samples, and its expression did not correlate with tumor grade. The sensivity and specificity were 80% and 87%, respectively (39).

Aurora A kinase (AURKA) is a cell-cycle associated kinase that regulates mitosis and is expressed in BC tissue samples. It was already linked to BC progression and correlates with stage, grade and prognosis. It also has a good performance in discriminating low-grade BC from normal urothelium, by the Aura Tek FDP TestTM in urine, which can detect BC recurrence, especialy in patients with haematuria. The sensitivity and specificity of AURKA were 83.6 and 65.2%, respectively (40, 41).

Some glycoproteins can be found in low and high-grade NMIBC. Sixty-three glycoproteins were exclusively identified in cancer samples compared with healthy controls, such as increased expression of urinary stem-cell marker CD44, which was associated with high-grade MIBC and poor prognosis (42).

The analysis of panels of urinary proteins is also an interesting approach. Previous studies from Theodorescu et al. (2006), Schiffer et al. (2009) and Frantzi et al. (2016) have already demonstrated that this approach may increase the sensitivity of the BC diagnosis, although the specificity range is still suboptimal (43-45) (Table-1). Chen et al. have found that the combination of decreased levels of epidermal growth factor (EGF), and increased levels of serum amyloid A-4 protein (SAA4) had higher diagnostic capacity in discriminating BC from hernia patients than either marker alone (46). Members of the serum amyloid A (SAA) family of acute-phase proteins are expressed in inflammation, but the knowlodge of alterations of SAA4 levels in cancer is still limited. The cytoplasmic domain of the human proEGF transmembrane region was reported as a novel suppressor of human thyroid carcinoma cell motility and cathepsin L-mediated elastinolytic invasion (47, 48).

The iTRAQ (isobaric tag for relative and absolute quantitation) technique was also applied to discover proteins with differential levels between pooled urine samples from controls and BC patients with different grades/stages, and the results revealed 55 proteins elevated in BC patients; Western blot confirmed the levels of apolipoprotein A-I (APOA1), apolipoprotein A-II (APOA2), heparin cofactor 2 (SERPIND1) precursor and peroxiredoxin-2 (PRDX2) were significantly higher in BC samples. Commercial ELISA for APOA1 confirmed its potential value for diagnosis (94.6% sensitivity, 92.0% specificity) and early detection (83.8% sensitivity and 94.0% specificity). Later, the same group used Liquid Chromatography Multiple Reaction Monitoring-Mass Spectrometry (LC-MRM/MS) approach, and found 12 proteins with higher concentrations in the BC group than in the hernia and urinary tract infection/hematuria groups, generating a six-peptide marker panel (afamin - AFM, adiponectin - ADIPOQ, complement C4 gamma chain, APOA2 precursor, ceruloplasmin - CP, and prothrombin - F2). The AUC was 0.814, with a 76.3% positive predictive value, and a 77.5% negative predictive value (49, 50).

Besides the main goal of developing protein biomarkers for diagnosis of primary tumor and its surveillance, the distinction of NMIBC and MIBC is also of great interest, because it can dramatically change the management of the disease. Survivin is an anti-apoptotic protein correlated with bladder cancer diagnosis, higher tumor grade and stage, that is also a predictor of invasiveness (51-53). Another prospective study used mass spectrometry to identify a panel of proteins able to discriminate MIBC from NMIBC, which were then validated in additional samples from healthy volunteers, and patients with malignant and nonmalignant genitourinary conditions. Four sequenced polypeptides (uromodulin - UMOD, collagen alpha-1(I) chain - COL1A1, collagen alpha-1(III) chain - COL3A1, and membrane-associated progesterone receptor component 1 - PGRMC1) formed a panel predictive of MIBC, with sensitivity of 81% and specificity of 57%. A model including tumor grade and panel polypeptide levels improved sensitivity to 92% and specificity to 68% (54).

In addition to evaluating proteins diluted in urine, proteins confined to urinary exosomes are also of great interest. Exosomes are small extracellular vesicles (EVs) with a significant role in different steps of cancer development. These EVs are present in nearly all human body fluids, including urine, and particularly enriched in tumor microenvironment, making them a field of big interest and research for BC biomarkers. Alterations in the content of EVs in BC, particularly in genetic material and protein levels were previously observed (55). Looking specifically to proteins, we have some examples. The tumor-associated calcium-signal transducer 2 (TACSTD2), a cell-surface glycoprotein, is elevated in BC exosomes; TACSTD2 concentrations measured by LC-MRM/MS were 6.5-fold higher in BC urinary EVs than in hernia urinary EVs (p=0.02, AUC=0.735) (56). Furthermore, EVs purified from the urine of high-grade BC contained significantly higher EGF-like repeat and discoidin I-like domain-containing protein 3 (EDIL-3) levels than exosomes from the urine of healthy controls. EDIL-3 is an extracellular matrix glycoprotein released from endothelial cells and mediates endothelial cell attachment and migration; it is also related to BC progression (57). Alpha-1-antitrypsin (SERPIN1) and Histone H2B type 1-K (HIST1H2BK) were found in high levels in urinary EVs, and when HIST1H2BK is present, it elevates to 3-fold higher the chance of recurrence (58). Increased Periostin (POSTN)-rich EVs demonstrated higher chance of progression and poor prognosis (59).

### Tissue proteomics

Although urinary proteomic studies may provide valuable insights into tumor biology, this information relates only indirectly to the tumor microenvironment, via its secreted proteins. Thus, the analysis of urine proteome does not allow a full description of the molecular alterations underlying BC. In this context, the direct investigation of proteins present in BC tissues may improve our understanding of the tumor metabolism, growth and invasiveness in a more direct and in-depth manner. Moreover, it may enhance the understanding about the relationship between tumor and urinary BC proteomes. Table-2 summarizes the most promising novel tissue protein biomarkers.

**Table 2:** Potential tissue protein biomarkers in Bladder Cancer.

Protein	Subjects	Context	Results
Profilin-1 (PFN1) Zoidakis et al., 2012 (60)	35 patients with stage pTa-pT2 + cancer	Prediction of invasiveness	Decreased PFN1 expression in invasive (T2) versus high risk non-invasive (T1G3) tumors, correlation with poor prognosis and increased mortality
Histone H2B (H2B) and Zinc finger protein 335 (NIF-1) Frantzi et al., 2013 (61)	32 patients with stage pTa-pT2+	Prediction of progression	Potential marker for discriminating BC stages, association with tumor progression
Bladder cancer-associated protein (BLCAP) Moreira et al., 2010 (64)	120 bladder specimens: histologically normal or various tumor stages	Prediction of progression, prognostic value	Loss of BLCAP expression confers a poorer prognosis for patients
Cystatin B (CSTB) Feldman et al., 2009 (63)	37 BC and 35 normal urothelial specimens	Prediction of recurrence and progression	Increased CSTB expression correlated with stage and grade, recurrence and progression
Maspin Kramer et al., 2010 (66)	162 patients with stages pTa-T1 cancer (NMIBC)	Prediction of recurrence and progression	Low Maspin expression correlated to tumor progression and recurrence
Carbonic anhydrase 9 (CAIX) Klatte et al., 2009 (67)	340 patients with BC of all stages	Prediction of recurrence, progression and survival	For NMIBC, higher CAIX was associated with poorer recurrence-free survival. In MIBC patients who underwent cystectomy, higher CAIX related to worse overall survival
Cyclin D1 (CCND1) Seiler et al., 2014 (68)	152 patients who underwent radical cystectomy	Response to adjuvant chemotherapy and survival	High CCND1 levels associated with disease-specific survival in patients treated with adjuvant chemotherapy
TP53, p21, p27kip1 and p107 Shariat et al., 2012 (69)	324 BC patients with pT1-T2 who received radical cystectomy	Prediction of recurrence and survival	Increased prediction accuracy for disease recurrence and cancer specific mortality by 15.6% and 14.8%, respectively (p <0.001)
Cathepsin E, Maspin, PLK1, survivin Fristrup et al., 2012 (70)	693 patients with stages pTa-T1 cancer (NMIBC)	Prediction of progression	Expression of cathepsin E, maspin, Plk1, and survivin significantly associated with progression to stage T2-T4 bladder cancer
Androgen receptor, JMJD2A and LSD1-AR Kauffman et al., 2011 (71)	72 patients who underwent radical cystectomy	Prediction of progression and survival	Significant reduced expression associated with cancer stage progression, muscle invasion and lymph node metastasis

A great number of studies has tried to correlate the urinary proteome with tissue proteome in BC. This is a very difficult task, because BC is highly heterogeneous, and the individual molecular profile has a significant impact on the disease outcome. Some examples of these proteins are profilin-1 (PFN1), zinc finger protein 335 (NIF-1), histone H2B (H2B), phosphoglycerate mutase 1 (PGAM1), cystatin B (CSTB), and bladder cancer-associated protein (BLCAP).

PFN1 belongs to the family of profilins which are small actin-binding proteins regulating the dynamics of actin polymerization and cell motility. This protein was found to be differentially expressed in the urine of MIBC patients, compared to NMIBC and benign controls, with high interindividual variability. Using a tissue microarray analysis, a marked decrease of PFN1 expression was observed in the epithelial cells of invasive (T2) versus high risk non-invasive (T1G3) tumors, which was strongly correlated with poor prognosis and increased mortality (60).

H2B (protein associated with DNA damage response) and NIF-1 (protein involved in histone methyltransferase activity and in nuclear receptor-mediated transcription) were analyzed in urine samples by ELISA, and in BC tissue by immunohistochemistry; results support changes in expression of both proteins in tumor progression, denoting a potential marker for discriminating BC stages (61).

Another study of urinary and tissue correlation found PGAM1, a vital enzyme in glycolytic pathway that catalyzes the conversion of 3-phosphoglycerate to 2-phosphoglycerate, to be important in tumorigenesis, invasion and metastasis of BC. Immunohistochemical data showed increased expression of PGAM1 in BC tissue correlated with severity of histological grade. The downregulation of PGAM1 upregulated its substrate 3-phosphoglycerate, with consequent downregulation of 2-phosphoglycerate, inhibiting aerobic glycolysis and oxidative pentose phosphate pathway (oxPPP), an essential mechanism to cancer cell proliferation. These findings suggest PGAM1 might be a promising therapeutic target (62).

CSTB is an inhibitor of cathepsin proteases. Cathepsin proteases seem to be increased in cancer, and the balance of the protease/inhibitor axis may play an important role. Increased expression of cystatin B in BC tissue correlated with stage and grade, recurrence and progression (63).

BLCAP is an 87-amino acid, evolutionarily conserved protein, with an unknown cellular function. Its expression and cellular localization were studied in benign bladder urothelium and urothelial carcinomas. Interestingly, BC patients were categorized into four groups based on expression levels and subcellular localization of BLCAP; the increased expression of this protein confers a poorer prognosis for patients (64). Tissue-based molecular markers can have prognostic value not only in BC, but also in upper tract urothelial carcinoma (UTUC) (65).

Maspin is a protein of the family of serine protease inhibitors that participates in angiogenesis and metastasis in tumor tissues; in a study with 162 patients with stages pTa-T1 NMIBC, maspin expression predicted progression with a sensitivity of 95% and a specificity of 70% (p <0.001); and recurrence, with a sensitivity of 52% and specificity of 67% (p <0.05) (66).

Carbonic anhydrase 9 (CAIX) was investigated in 340 patients with BC of all stages. All normal urothelial tissue samples were negative for CAIX expression, whereas 71% of BC expressed CAIX. For NMIBC, higher CAIX expression was associated with poorer recurrence-free survival and, for T1 tumors, a 6.5-fold higher risk of progression into MIBC. In patients who underwent cystectomy, higher CAIX expression was associated with worse overall survival (67).

Cyclin D1 (CCND1) is an important promoter of the cell cycle, whose amplification status was evaluated by fluorescence in situ hybridization and immunohistochemistry on tissue microarrays from 152 lymph node-positive urothelial BC treated by cystectomy and lymphadenectomy. CCND1 amplification status and CyclinD1 expression were independent risk factors in BC metastasis, and high nuclear CyclinD1 expression in lymph node metastases predicted favorable response to chemotherapy (68).

Combinations of markers may also have accurate prediction. Cell cycle related proteins (p53, pRB, p21 and p27) were tested in 324 BC patients with pT1-T2 who received radical cystectomy. The results showed increased prediction accuracy for disease recurrence and cancer specific mortality by 15.6% and 14.8%, respectively (p <0.001) (69).

Another study evaluated the protein expression of cathepsin E, maspin, polo-like kinase 1 (Plk1), and survivin in 693 patients with stages pTa-T1 NMIBC; these proteins were found to be significantly associated with progression to stage T2 to T4 cancer (70).

The expression of Androgen receptor (AR), JMJD2A and LSD1 (recently discovered AR coregulator proteins that mediate AR-dependent transcription) were analysed in 72 radical cystectomy specimens by immunohistochemistry. A significant reduction in all three proteins occurred with cancer stage progression, muscle invasion, extravesical extension, and lymph node metastasis (71).

A recent study stratified three groups of NMIBC tumors based on the tissue proteomic pattern, named NMIBC Proteomic subtypes (NPS). The first group (NPS1), with predominant pathology of high stage and grade tumors, overexpresses proteins related to immune and inflammatory reactions, cell proliferation and DNA damage response; the second group (NPS2) presented with mixed pathology tumors, and proteins implicated on epithelial to mesenchymal transition; finally, the third group (NPS3) presented predominant pathology of low stage and grade tumors, with luminal and differentiation markers. NPS1 had the closest proteomic profile of MIBC (72).

### Blood proteomics

Whole blood is collected in a minimally invasive manner, and presents a more complex proteomic profile, which may lead to lower sensitivity (73). The analysis of changes in the serum protein profile of BC patients can reveal the complex interplay between tumor tissue and the surrounding vascular microenvironment. Proteomic studies using blood are scarce in comparison to urine samples (74).

Bansal et al. described five differentially expressed proteins in BC, of which two (Protein S100-A8 - S100A8 and Protein S100-A9 - S100A9) were accurately capable (AUC 0.94) of differentiating BC from healthy controls (75). Schwamborn et al. have studied the proteomic patterns of serum samples from BC patients compared to controls, achieving 96.4% of sensitivity and 86.5% of specificity in diagnosis of BC (76). Lee et al. found that plasma proteins involved in inflammatory responses were upregulated, while proteins of cytoskeleton and cytoskeletal regulation were downregulated (77).

### Cell lines proteomics

Cell lines originated from BC are essential tools for basic research, since they can offer the access to mutations and tumor biology without the need to directly access BC tissue (78, 79). A recent systematic review described 127 human BC cell lines, out of which 103 have available molecular data, and 69 were profiled by at least one “omic” technology. The most frequently characterized cell lines are HT-1197, T24, and TCCSUP. Only a small number of these human BC cell lines are of low stage (12/127) and low-grade (8/127) tumors, mainly due to the difficulty of culturing low-grade tumors in vitro, although low-grade tumors represent the vast majority of clinical BC (80).

Proteomic studies using BC cell lines have already shown some interesting findings. Welton et al. examined EVs from HT1376 BC cell line, and identified 353 proteins, with elevated levels of Platelet glycoprotein 4 (CD36), CD44 antigen (CD44), Trophoblast glycoprotein (TPBG), basigin (BSG), and 5’-nucleotidase (NT5E) (81). Beckham et al. showed that EVs secreted from high-grade BC cell lines leaded to angiogenesis and migration of tumor and endothelial cells, mediated by EDIL3 protein (57). The proteomics of T24 BC cell line identified overexpression of cullin-3 (CUL3), a protein involved in ubiquitination, whose silencing leads to decreased cell proliferation and migration. This protein was associated with higher tumor stage, agressiviness and metastasis (82).

The upregulation of hypoxia-related proteins was also seen on three different BC cell lines (T24, 5637 and HT1376), for example, for hypoxia-inducible fator 1-alpha (HIF1A) and its transcriptionally-regulated protein carbonic anhydrase 9 (CA9). Increased levels of lactate biosynthesis were also observed, indicating anaerobic metabolism, associated with enhanced epithelial to mesenchymal transition features. The interesting fact is that these effects were reversed by reoxygenation, mediated by glycoproteins, opening a potential pathway to target treatment studies in future (83).

## CONCLUSIONS

The analysis of molecular profiling data reveals that BC is highly heterogeneous, but this detailed molecular characterization seems to be a good strategy to establish better diagnostic, follow-up and treatment interventions, towards an increasingly personalized medicine (8, 84). Novel biomarkers can also decrease the economic burden associated with invasive procedures, frequently associated with hospitalization and complications. Panels of biomarkers may be an alternative to increase the sensitivity of the tests for BC diagnosis. Although the currently studied protein markers have shown promising value in diagnosis, surveillance and prognostic of BC, prospective clinical trials are needed to validate these proteomic data, with a much bigger challenge to translate its potential to the clinical practice.

## ABBREVIATIONS

BC: bladder cancer
NMIBC: non-muscle invasive bladder cancer
MIBC: muscle invasive bladder cancer
NUMA1: nuclear mitotic apparatus protein 1
NMP22: nuclear matrix protein-22
FDA: Food and Drug Administration
KRT8, KRT18, KRT20: cytokeratin 8, cytokeratin 18, cytokeratin 20
RT: reverse transcription
PCR: polimerase chain reaction
BLCA-1 and BLCA-4: bladder cancer markers 1 and 4
AURKA: aurora A kinase
EGF: epidermal growth factor
SAA4: serum amyloid A-4 protein
AUC: area under the ROC curve
iTRAQ: isobaric tag for relative and absolute quantitation
APOA1, APOA2: apolipoprotein A-I, apolipoprotein A-II
SERPIND1: heparin cofactor 2
PRDX2: peroxiredoxin-2
LC-MRM/MS: liquid Chromatography Multiple Reaction Monitoring-Mass Spectrometry
EVs: extracellular vesicles
TACSTD2: tumor-associated calcium-signal transducer 2
EDIL-3: EGF-like repeat and discoidin I-like domain-containing protein 3
PFN1: profilin-1
NIF-1: zinc finger protein 335
H2B: histone H2B
PGAM1: phosphoglycerate mutase 1
CSTB: cystatin B
BLCAP: bladder cancer-associated protein
NPS: NMIBC ProteomicSubtypes
